# Preclinical animal models of COPD

**DOI:** 10.1186/1476-9255-10-S1-P25

**Published:** 2013-08-14

**Authors:** Ross Vlahos

**Affiliations:** 1 Department of Pharmacology, The University of Melbourne, Parkville, Victoria, 3010, Australia

## Introduction

COPD is a complex inflammatory airway disease characterized by airflow limitation that is not fully reversible. The mechanisms and mediators that drive the induction and progression of chronic inflammation, emphysema and altered lung function are poorly understood. Current treatments have limited efficacy in COPD and there is a clear need for new therapies that can prevent the induction and progression of COPD. Animal modeling systems that accurately reflect disease pathophysiology continue to be essential to the development of new therapies.

## Aim

To develop preclinical *in vivo* models that recapitulate many of the features of COPD.

## Materials and methods

Mice were exposed to cigarette smoke (CS) for up to 6 months and the hallmarks of COPD investigated. In some experiments, CS-exposed mice were infected with influenza A virus (H3N1, Mem71 strain) to mimic acute exacerbations of COPD (AECOPD).

## Results

We have developed preclinical murine models of COPD that replicate key clinical traits observed in human disease, including BAL and lung inflammation, induction of various cytokines/chemokines/proteases, increased oxidative stress, goblet cell hyperplasia, emphysema and lung function deterioration. These models have identified novel pathways that control leukocyte accumulation and lung damage. When modeling systemic comorbidities of COPD, we found that mice exposed to CS had reduced body weight, fat mass, hind limb skeletal muscles mass, grip strength and aerobic endurance. CS altered the mRNA expression of a number of genes associated with the regulation of skeletal muscle mass including insulin-like growth factor-I, atrogin-1 and IL-6. Moreover, the levels of neuropeptide Y were reduced in CS-exposed mice. Compared to CS or influenza alone, mice exposed to CS and then influenza had more BAL inflammation, lung virus titres, altered profiles of key cytokines, worse lung pathology and more virus-specific, activated CD8+ T lymphocytes in BAL. CS exposure caused temporary weight loss and when smoking ceased after viral infection, CS and influenza mice regained significantly less weight than CS alone mice.

## Conclusions

Using these preclinical *in vivo* models of COPD, we have identified key mediators which initiate and drive COPD. Understanding the neuromolecular and systemic processes that lead to loss of body weight in smokers may provide novel intervention points for improving cessation therapy and may also contribute to understanding wasting syndromes in smoking-related lung diseases. With respect to exacerbations of COPD, it is clear that CS exposure worsens the host response to influenza virus.

